# Can photobiomodulation therapy be an alternative to pharmacological therapies in decreasing the progression of skeletal muscle impairments of *mdx* mice?

**DOI:** 10.1371/journal.pone.0236689

**Published:** 2020-08-12

**Authors:** Shaiane Silva Tomazoni, Heliodora Leão Casalechi, Cheila de Sousa Bacelar Ferreira, Andrey Jorge Serra, Humberto Dellê, Rodrigo Barbosa de Oliveira Brito, Brunno Lemes de Melo, Adriane Aver Vanin, Neide Firmo Ribeiro, Amanda Lima Pereira, Kadma Karênina Damasceno Soares Monteiro, Rodrigo Labat Marcos, Paulo de Tarso Camillo de Carvalho, Lucio Frigo, Ernesto Cesar Pinto Leal-Junior

**Affiliations:** 1 Department of Global Public Health and Primary Care, Physiotherapy Research Group, University of Bergen, Bergen, Norway; 2 Laboratory of Phototherapy and Innovative Technologies in Health (LaPIT), Universidade Nove de Julho (UNINOVE), São Paulo, São Paulo, Brazil; 3 Postgraduate Program in Medicine, Universidade Federal de São Paulo (UNIFESP), São Paulo, São Paulo, Brazil; 4 Postgraduate Program in Medicine, Universidade Nove de Julho (UNINOVE), São Paulo, São Paulo, Brazil; 5 Postgraduate Program in Rehabilitation Sciences, Universidade Nove de Julho (UNINOVE), São Paulo, São Paulo, Brazil; 6 Postgraduate Program in Biophotonics Applied to Health Sciences, Universidade Nove de Julho (UNINOVE), São Paulo, Brazil; 7 Department of Periodontology, Dental Research Division, Universidade de Guarulhos (UnG), Guarulhos, São Paulo, Brazil; VA Loma Linda Healthcare System, UNITED STATES

## Abstract

**Objective:**

To compare the effects of photobiomodulation therapy (PBMT) and pharmacological therapy (glucocorticoids and non-steroidal anti-inflammatory drugs) applied alone and in different combinations in *mdx* mice.

**Methods:**

The animals were randomized and divided into seven experimental groups treated with placebo, PBMT, prednisone, non-steroidal anti-inflammatory drug (NSAIDs), PBMT plus prednisone and PBMT plus NSAID. Wild type animals were used as control. All treatments were performed during 14 consecutive weeks. Muscular morphology, protein expression of dystrophin and functional performance were assessed at the end of the last treatment.

**Results:**

Both treatments with prednisone and PBMT applied alone or combined, were effective in preserving muscular morphology. In addition, the treatments with PBMT (p = 0.0005), PBMT plus prednisone (p = 0.0048) and PBMT plus NSAID (p = 0.0021) increased dystrophin gene expression compared to placebo-control group. However, in the functional performance the PBMT presented better results compared to glucocorticoids (p<0.0001). In contrast, the use of NSAIDs did not appear to add benefits to skeletal muscle tissue in *mdx* mice.

**Conclusion:**

We believe that the promising and optimistic results about the PBMT in skeletal muscle of *mdx* mice may in the future contribute to this therapy to be considered a safe alternative for patients with Duchenne Muscular Dystrophy (DMD) in a washout period (between treatment periods with glucocorticoids), allowing them to remain receiving effective and safe treatment in this period, avoiding at this way periods without administration of any treatment.

## Introduction

Duchenne muscular dystrophy (DMD) is a rare, severe and progressive neuromuscular disease [1] caused by a mutation in the dystrophin gene, lead to a deficiency in the production of dystrophin [2]. The essential function of dystrophin in the muscle is stabilizes the fibers during eccentric muscle contraction [3]. The loss of this stabilization leads to myofibers become more susceptible to contraction-induced injury [4], a progressive muscle fibers wasting and replacement by fat and connective tissue [2], compromising the regeneration process [5].

Animal models are available for the development of innovative therapies for the treatment of DMD [6]. The *mdx* mouse (C57BL/10ScSn-DMD^*mdx*^/J) is a preclinical model that has absence of full-length dystrophin expression [6–8], resulting in dystrophic symptoms similar to that seen in humans with DMD [8]. In *mdx* mice is observed morphological changes indicative of a degenerative process of skeletal muscle tissue, such as fibrosis, decreased number and size of muscle fibers and clustering of nuclei in the center of muscle fibers [9, 10]. Moreover, muscle fiber degeneration is accompanied by immune and inflammatory responses [11]. Lastly, *mdx* mice also present decreased of functional performance [10].

Besides severity, currently there is no cure for DMD [12]. However, there are many different kinds of treatments available trying to decrease its’ progression and symptoms [13]. Exhaustive clinical management and glucocorticoids treatment have improved outcomes in patients with DMD [14, 15]. There is evidence that glucocorticoid treatment improves short-term muscle strength and function [14], delays the respiratory problems and development of cardiac complications [1, 16, 17]. However, long-term glucocorticoid therapy is associated with serious adverse effects [14]. In addition to the use of glucocorticoids, there is some indication that treatment with non-steroidal anti-inflammatory drugs (NSAIDs) has beneficial effects under the morphology of *mdx* mouse, pointing to reduction the progression of DMD [11]. However, prolonged use of these drugs also leads to the development of important adverse effects [18]. On the other hand, recent research has demonstrated that the use of PBMT can also delay the progression of the DMD, with protective effect on skeletal muscle of *mdx* mice, with the advantage of does not causing adverse effects to date [9, 10, 19].

PBMT is a non-pharmacological and non-thermal intervention that uses non-ionized forms of light (low-level laser, light emitting-diodes and broadband light) to promote modulation of inflammation, tissue regeneration and pain relief [20]. The use of PBMT to manage DMD is a novel area of research, however studies have shown that PBMT works by increasing cell proliferation and accelerating cell differentiation in *in vitro* primary culture of skeletal muscle dystrophic cells [19], and reducing inflammatory and oxidative stress in *mdx* mouse both *in vitro* [19] and *in vivo* studies [9, 21]. Furthermore, the preventive use of PBMT decreases the skeletal muscle damage and fatigue also in *mdx* mice [9, 21].

Although some effects of PBMT have already been demonstrated in *mdx* mice, to date there are no studies comparing PBMT with glucocorticoids, the first line treatment adopted in DMD patients, and NSAIDs that could be a pharmacological alternative. Therefore, we performed this study aiming to compare the effects of PBMT and pharmacological therapy (glucocorticoids and NSAIDs) applied alone and in different combinations, on muscular morphology, protein expression of dystrophin and functional performance of *mdx* mice.

## Methods

### Animals

A total of 5 Wild type (C57BL/10ScSn) mice and 85 *mdx* mice from the central animal facility of the Nove de Julho University (UNINOVE) were used. The animals were kept under standard conditions of temperature (22 to 24 °C), relative humidity (40 to 60%), a 12-hour light-dark cycle, and provided water and feed *ad libitum*. All experimental protocols were submitted and approved by the Animal Experimentation Ethics Committee of the University of Nove de Julho (UNINOVE) (Protocol AN008.2014) and were performed in accordance with the standards of the Brazilian College of Animal Experimentation (COBEA).

### Experimental groups

The animals were randomized and divided into seven experimental groups as described below:

- WT (wild type) group (C57BL/10ScSn): Five wild type mice were not subjected to any procedure or treatment. These five animals were used in protein expression analysis. The data/images regarding histological analysis from this group were taken from a previous experiment [10], however, the experiments were carried out at the same time than the other experimental groups of this study.

In the following groups all animals used were *mdx* mice:

- Placebo-control group: Ten *mdx* mice were treated with placebo-control PBMT (using a placebo PBMT probe). Five animals were used in protein expression analysis, and five animals were used in functional performance analysis. The data/images regarding histological analysis from this group were taken from a previous experiment [10], however, the experiments were carried out at the same time than the other experimental groups of this study.
- PBMT group: Fifteen *mdx* mice were treated with PBMT with dose of 10 J [10]. Five animals were used in morphological analysis, five in protein expression analysis, and five animals were used in functional performance analysis.
- Prednisone group: Fifteen *mdx* mice were treated with oral prednisone (glucocorticoid) with dose of 1.5 mg/kg/day [22]. Five animals were used in morphological analysis, five in protein expression analysis, and five animals were used in functional performance analysis.
- NSAID group: Fifteen *mdx* mice were treated with oral ibuprofen (NSAID) with dose of 25 mg/kg/day [11]. Five animals were used in morphological analysis, five in protein expression analysis, and five animals were used in functional performance analysis.
- PBMT + Prednisone group: Fifteen *mdx* mice were treated with the combination of PBMT (with dose of 10 J) [10] and oral prednisone (with dose of 1.5 mg/kg/day) [22]. Five animals were used in morphological analysis, five in protein expression analysis, and five animals were used in functional performance analysis.
- PBMT + NSAID group: Fifteen *mdx* mice were treated with the combination of PBMT (with dose of 10 J) [10] and oral ibuprofen (with dose of 25 mg/kg/day) [11]. Five animals were used in morphological analysis, five in protein expression analysis, and five animals were used in functional performance analysis.

All treatments started with animals at 6 weeks of age.

### Treatments

All treatments were performed during 14 consecutive weeks:

#### Single therapies

*Administration of pharmacological therapy*. Pharmacological therapy with both glucocorticoid and NSAID was administered via water bottles containing the required dose of the drug. The total drug concentration available in each bottle of water was calculated from the average daily water consumption per animal. For this determination, a high volume of water (500 ml) was provided per cage (with 5 animals in cage) and after one week the volume of water remaining in each cage was observed. The difference between the water volume at the beginning and at the end of the week determined the water consumption of 5 animals for 7 days. This observation was repeated over 4 weeks to make the average daily water consumption per animal.

- Administration of glucocorticoid (Prednisone): Pharmacological therapy with glucocorticoid (Prednisone) was performed according to a previous study carried out by Janssen et al. [22]. Prednisone dosage was calculated by an allometric scaling of drugs clinical dosages for use in animals (FDA Guide—Guidance for industry estimating the maximum safe starting dose in initial clinical trial for therapeutics in adult healthy volunteers). Water bottles containing prednisone (Sigma-Aldrich, USA) with dose of 1.5mg/kg/day were made available to the animals every day for 14 consecutive weeks.
- Administration of NSAID: The dose of NSAID (Ibuprofen) was based on the study of Serra et al. [11] and has been established in order to achieve therapeutic effects minimizing the occurrence of undesirable adverse effects. Water bottles containing ibuprofen (Sigma-Aldrich, USA) with dose of 25 mg/kg/day were made available to the animals every day for 14 consecutive weeks.

*Application of active PBMT*. The PBMT parameters used were based on the best dose found on the study by Albuquerque-Pontes et al. [10]. A cluster probe with 9 diodes (1 laser diode of 905 nm, 4 LED diodes of 875 nm, and 4 LED diodes of 640 nm—manufactured by Multi Radiance Medical^™^) was used. Only one point on the ventral region of the animal’s tibialis anterior muscle (bilaterally) was irradiated. To irradiate the animals, the spot was kept in direct contact with the animal’s skin, applying light pressure on the tissue. The same technology has previously demonstrated to do not promote significant increases in skin temperature (less than 1°C) even when doses of 50 J were irradiated in individuals with dark skin [23]. The full description of PBMT parameters is summarized in Table 1. The active PBMT was administered once daily, 3 times a week (on alternate days) for 14 consecutive weeks. The optical power of the device was verified at every 2-week period by a researcher that was not involved in data collection and analysis, for such it was employed a Thorlabs^®^ power meter (Model S322C, Thorlabs^®^, Newton, NJ, USA).

**Table 1:** Parameters for photobiomodulation therapy (PBMT).

Number of lasers	1 Super-pulsed infrared
Wavelength (nm)	905 (±1)
Frequency (Hz)	250
Peak power (W)—each	25
Average mean optical output (mW)—each	0.625
Power density (mW/cm^2^)—each	1.42
Energy density (J/cm^2^)—each	0.11
Dose (J)—each	0.048
Spot size of laser (cm^2^)—each	0.44
Number of red LEDs	4 Red
Wavelength of red LEDs (nm)	640 (±10)
Frequency (Hz)	2
Average optical output (mW)—each	15
Power density (mW/cm^2^)—each	16.66
Energy density (J/cm^2^)—each	1.283
Dose (J)—each	1.155
Spot size of red LED (cm^2^)—each	0.9
Number of infrared LEDs	4 Infrared
Wavelength of infrared LEDs (nm)	875 (±10)
Frequency (Hz)	16
Average optical output (mW)—each	17.5
Power density (mW/cm^2^)—each	19.44
Energy density (J/cm^2^)—each	1.497
Dose (J)—each	1.3475
Spot Size of LED (cm^2^)—each	0.9
Magnetic Field (mT)	35
Irradiation time per site (sec)	77
Total dose per site (J)	10.0
Total dose applied in muscular group	10.0
Aperture of device (cm^2^)	0.197
Device power density (mW/cm^2^)	663.07
Device energy density (J/cm^2^)	50.76
Application mode	Cluster probe held stationary in skin contact with a 90-degree angle and slight pressure

*Application of placebo PBMT*. Placebo PBMT was delivered using the same device that active PBMT, but without any emission of therapeutic dose. Animals were receive a total dose of 0 J in placebo mode. The placebo PBMT group was treated in the same way as the active PBMT group.

#### Combined therapies

All combined therapies were applied similarly and with the same parameters and dose used as when the therapies were applied alone.

### Collection of biological material

Twenty-four hours after the last therapy session the animals were anesthetized with a mixture of Ketamine and Xylazine (90 mg/kg and 10 mg/kg, respectively; König, Avellaneda, Argentina), administered intraperitoneally. Subsequently, the tibialis anterior muscle was surgically removed bilaterally and processed for further analysis of histology and Western Blott. After collection of the muscles the animals were euthanized (at 20^th^ week of age) with an overdose of a mixture of Ketamine and Xilazin (300 mg/kg and 30 mg/kg, respectively). The death of animals was confirmed through assessment of absence of heart beat for five minutes (palpation of cardiac area), and absence of pupillary response to light.

### Outcomes

All analysis were performed by a blinded researcher:

#### Histomorphometric analysis

The tibialis anterior muscles of the hind paw were collected and stored in 10% buffered formalin for histological processing; then, hematoxylin and eosin staining were performed in a routine method. Slides were photographed (Eclipse E-200; Nikon, Tokyo, Japan), the images of all groups were obtained using the 400x magnification. The images were presented with a similar photographic pattern. The morphology of the skeletal striated muscle tissue was analyzed using randomly 5 slides with 1:10 semi-serial sections from each animal. Three microscopic fields were randomly selected and photographed in 400x magnification to ImageJ software (National Institutes of Health, Bethesda, MD, USA). Evaluation was carried out by a blinded researcher. Number of fibers, number of centronuclear fibers and number of cluster nuclei were simply counted. The muscle fibers size (diameter) demanded a fiber cross-section trace. Connective tissue area demanded the outline of extracellular space between muscle fibers in order to ImageJ software proceed with area calculation. The images for the WT and placebo-control groups were obtained from a previous study [10].

#### Protein expression analysis—Western blotting

Frozen samples of tibialis anterior muscles were homogenized using ice-cold lysis buffer and proteinase inhibitor cocktail. Lysates corresponding to 30 μg of protein were subjected to 10% SDS-PAGE. Separated proteins were transferred to PVDF membrane (Amersham Biosciences, NJ, USA) and transfer effectiveness was examined with 0.5% Ponceau S. After blocking with 5% non- fat dry milk for 2 h at room temperature, PVDF membranes were probed with Abcam (Cambridge, MA, USA) primary antibodies for rabbit anti-dystrophin (1:5000) in overnight incubation. Membranes were then washed five times with PBS and incubated for 1 h with horseradish peroxidase-conjugated anti-rabbit (1:20000; Zymed, CA, USA). Membranes were again washed five times with blocking buffer and then rinsed twice with PBS. Antibodies binding were detected by chemiluminescence reagents (Amersham Biosciences, NJ, USA), and images were captured using an Amersham Imager 600 system. Quantification of target proteins were normalized for the internal control glyceraldehyde 3-phosphate dehydrogenase.

#### Functional performance

Functional performance evaluation protocol consisted in the exercise of climbing stairs with the following dimensions (1 x 0.09m, distance between the steps of 0.5 cm and 80°) using the animal’s own body weight (the ladder ends at a platform measuring 0.18 x 0.18m). The protocol began with the familiarization of the animal with the exercise to teach it to climb the ladder. This familiarization consisted of three repetitions of climb to the top, with an interval of 60 seconds to rest and was performed in two sessions with a 24-hour interval between them [10]. When necessary, a slight clamp stimulus for the animal to start moving was applied, however the number of stimuli was limited to five. After this familiarization, the assessments were performed before the treatments begin and 24 hours after the last therapy session, in order to evaluate how many times each animal was able to perform the climb up the stairs (with or without clamp stimuli) until it to came to fatigue. Fatigue was considered when the mouse could no longer perform the activity.

### Statistical analysis

The data was tabulated and a normal distribution was determined through the Shapiro-Wilk test. Histomorphometric analysis and protein expression of dystrophin were analyzed using one-way ANOVA, followed by the Bonferroni post hoc test for multiple comparisons. For the functional analysis, the two-way ANOVA followed by the Bonferroni post hoc test was performed to verify statistical significance among groups. Data analysis was performed using the mean values and standard deviation (SD). However, in graphs, data are presented as mean and standard error of the mean (SEM). The level of statistical significance was set at p<0.05.

## Results

### Morphological analysis

**Fig 1:**
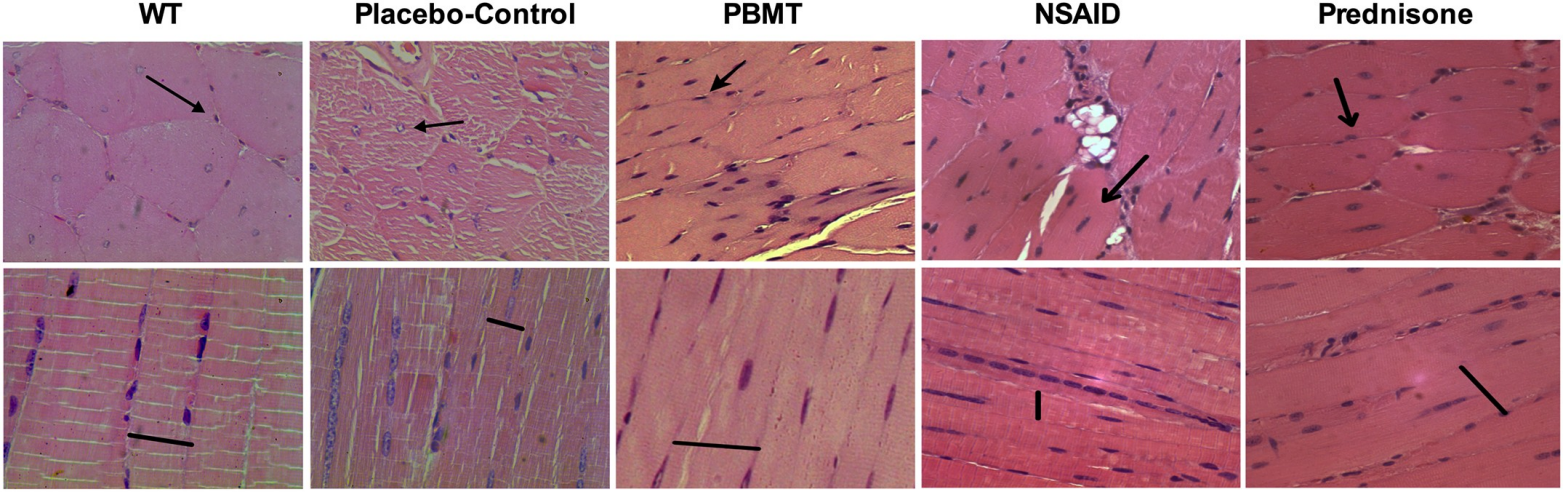
Photomicrographs of histological muscles sections (longitudinal and transversal sections) of WT, placebo-control, PBMT, NSAID and Prednisone groups (HE, original magnification x 400). Photomicrographs of WT and Placebo-control groups were obtained from a previous study [10].

**Fig 2:**
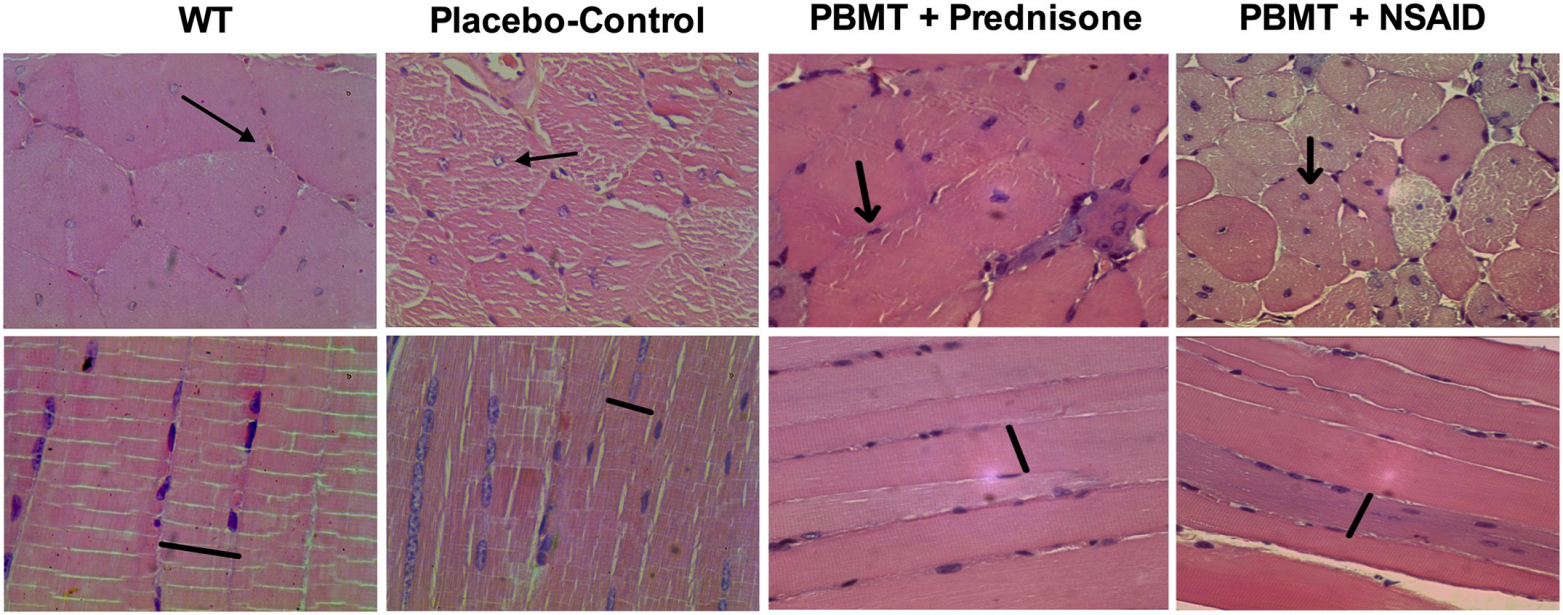
Photomicrographs of histological muscles sections (longitudinal and transversal sections) of WT, placebo-control, PBMT + Prednisone and PBMT + NSAID groups (HE, original magnification x 400). Photomicrographs of WT and Placebo-control groups were obtained from a previous study [10].

**Fig 3:**
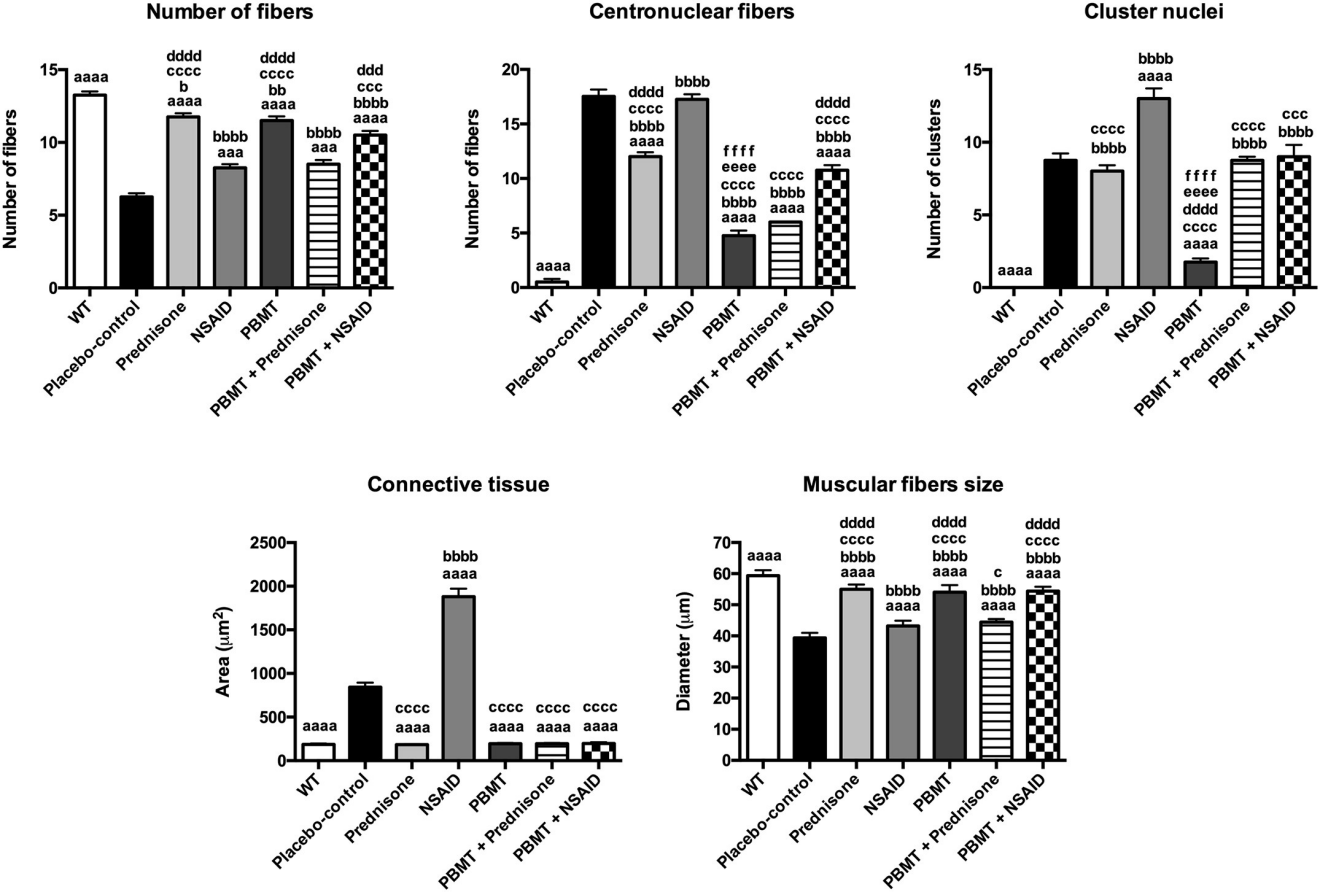
Histomorphometric analysis of all experimental groups. The ^aaa^ indicates a significant difference compared with Placebo-control group (p<0.001), ^aaaa^ indicates a significant difference compared with Placebo-control group (p<0.0001), ^b^ indicates a significant difference compared with WT group (p<0.05), ^bb^ indicates a significant difference compared with WT group (p<0.01), ^bbbb^ indicates a significant difference compared with WT group (p<0.0001),), ^c^ indicates a significant difference compared with NSAID group (p<0.05), ^ccc^ indicates a significant difference compared with NSAID group (p<0.001), ^cccc^ indicates a significant difference compared with NSAID group (p<0.0001), ^ddd^ indicates a significant difference compared with PBMT + Prednisone group (p<0.001), ^dddd^ indicates a significant difference compared with PBMT + Prednisone group (p<0.0001), ^eeee^ indicates a significant difference compared with Prednisone group (p<0.0001), and ^ffff^ indicates a significant difference compared with PBMT + NSAID group (p<0.0001).

The morphological analysis evidenced the dystrophic pattern of *mdx* mice (placebo-control group) as shown in Fig 1, and the changes observed in the other experimental groups as shown in Fig 2. Compared to wild-type group (WT), the placebo-control group showed signs of degeneration, such as statistically significant positioning of the nuclei at the center of the muscle fibers (p<0.0001), nuclear clusters (p<0.0001), and decrease in the number (p<0.0001) and size (p<0.0001) of muscle fibers. Furthermore, the placebo-control group presented a marked amount of connective tissue (p<0.0001) compared to WT group as shown in Fig 3. Regarding the treatments, the group treated with PBMT showed signs of improvement in all evaluated characteristics (p<0.0001) when compared to placebo-control group, and presented more consistent improvements when compared to the other treated groups (Figs 1, 2 and 3). Moreover, the group treated with NSAID (Figs 1 and 3) showed less alterations than the other treatments when compared with placebo-control group, with cell nuclei displaced to the center of the muscle fiber (p<0.0001), nuclear clusters (p<0.0001) and marked presence of connective tissue (p<0.0001). These findings demonstrate that NSAID was less effective than PBMT in reestablishing the morphological aspects unleashed through the disease. On the other hand, both Prednisone group (Figs 1 and 3) and the associated therapies (PBMT plus prednisone and PBMT plus NSAIDs) (Figs 2 and 3) showed some degree of minimization of the morphological aspects of the disease. However, in general this decrease was lower than observed in PBMT group. The quantitative analysis of main histological findings in different experimental groups is summarized in Fig 3.

### Protein expression analysis—Immunohistochemistry

**Fig 4:**
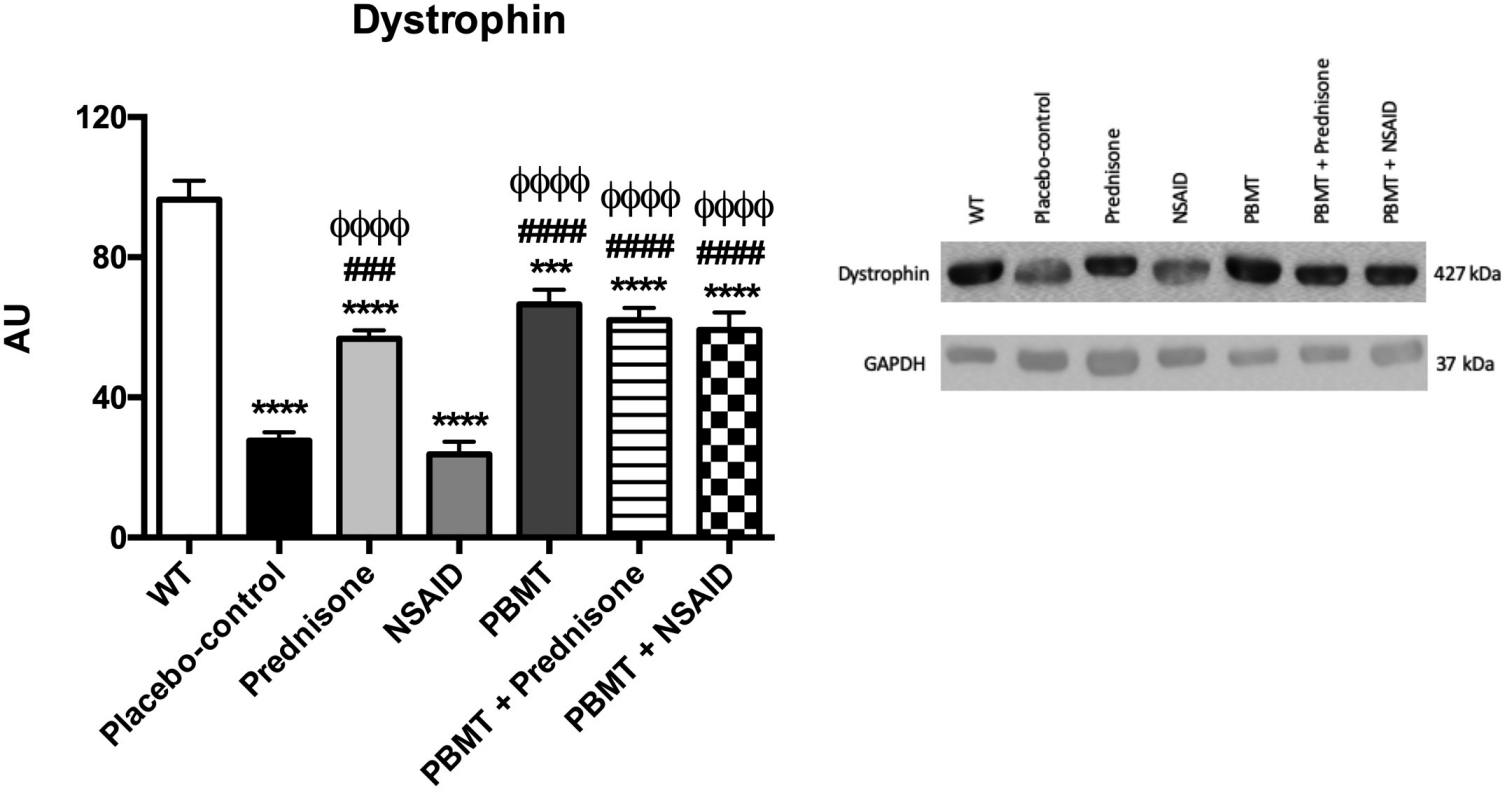
Protein expression of dystrophin in all experimental groups. The *** indicates a significant difference compared with WT group (p<0.001), **** indicates a significant difference compared with WT group (p<0.0001), ^###^ indicates a significant difference compared with placebo group (p<0.001), ^####^ indicates a significant difference compared with placebo group (p<0.0001), and ^ϕϕϕϕ^ indicates a significant difference compared with NSAID group (p<0.0001); n = 5 animals per group.

Fig 4 demonstrates the dystrophin protein expression of the animals from all experimental groups. We observed a statistically significant reduction of dystrophin protein expression in the placebo-control group (p<0.0001) and all other treatment groups (p<0.001 for PBMT group, and p<0.0001 for all other groups) compared to the WT group. Moreover, PBMT (p<0.0001), Prednisone (p<0.001), PBMT + Prednisone (p<0.0001) and PBMT + NSAID (p<0.0001) groups showed statistically significant increase in dystrophin gene expression compared to placebo-control group. In addition, Prednisone (p<0.0001), PBMT (p<0.0001), PBMT + Prednisone (p<0.0001), and PBMT + NSAID (p<0.0001) groups showed statistically significant increase in dystrophin gene expression compared to the NSAID group.

### Functional performance assessment

**Fig 5:**
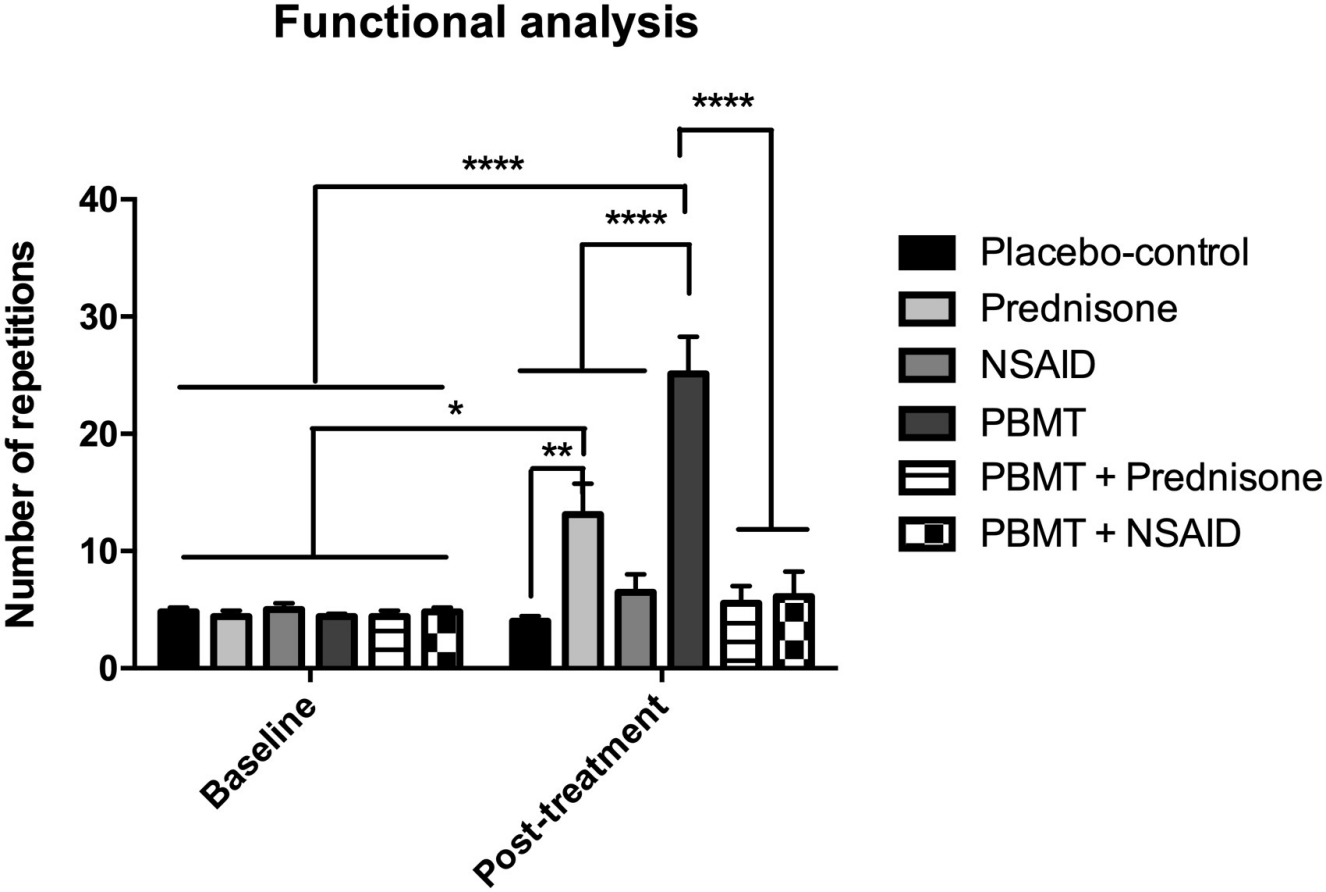
Functional performance assessment performed at baseline and post-treatment timepoints. The * indicates a significant difference compared with all experimental groups at baseline (p<0.05), ** indicates a significant difference compared to placebo-control group at post-treatment evaluation (p<0.01), and **** indicates a significant difference compared with all experimental groups at baseline, and all the other experimental groups at post-treatment evaluation (p<0.0001); n = 5 animals per group.

All experimental groups were homogenous prior to treatment (pre-treatment/baseline evaluation), indicating that meaningful comparisons between groups could be made. In the post-treatment evaluation, the Prednisone group showed a significantly increased number of repetitions when compared to Placebo-control group (p<0.01). Moreover, the PBMT group showed a significantly increased number of repetitions, both when compared to the Placebo-control group and to the other treatment groups (p<0.0001), as shown in Fig 5.

## Discussion

In this study we evaluated the effects of PBMT and pharmacological therapies applied alone on muscular morphology, protein expression of dystrophin and functional performance of *mdx* mice. In addition, we investigated whether the association of these therapies could enhance its isolated effects. For such, the characterization of the experimental model was initially performed through the comparison between *mdx* mice and wild type mice. As the experimental model demonstrated dystrophic characteristics (dystrophin reduction and morphological alterations) the *mdx* mice were treated with PBMT, prednisone or ibuprofen administrated alone or pharmacological therapies associated with PBMT. A limitation to our study is that a gene expression analysis of dystrophin was not performed, and therefore, must be considered in further projects.

Glucocorticoids and PBMT applied alone and in combination showed very similar results regarding the preservation of muscular morphology and increase of protein expression of dystrophin, suggesting that both treatments are effective for these outcomes. However, although the association between these two therapies was not detrimental it also did not add significant benefits. The treatment with NSAID applied alone showed only few signs of preservation of muscular morphology (increased number and size of muscular fibers), without effects on protein expression of dystrophin and in improvement of muscular function. Thus, it seems that NSAIDs is not a consistent alternative in *mdx* mice. On the other hand, the association between NSAID and PBMT showed better effects in both muscular morphology and protein expression of dystrophin, possibly due to the effects of PBMT and not necessarily due combined effects of both therapies. Regarding the functional analysis, it was observed that only *mdx* mice treated with glucocorticoid and PBMT presented increased performance. However *mdx* mice treated with PBMT alone had a greater increase in functional performance, evidencing the ergogenic effects of this therapy.

Glucocorticoids are considered gold standard treatment of DMD, due to their effect on the delay of the muscular symptoms related to disease progression [24]. Evidence suggests that prednisone has a protective effect on the accumulation of damaged fibers, presenting a lower amount of centronucleated muscle fibers [25–27] a characteristic corroborated in our study. Among the protective effects of glucocorticoids, the improvement in muscle regeneration by promoting satellite cell proliferation and increase in the density of myoblasts and myotubes [25–27] stand out. Clinically these effects contribute to delayed progression of muscle weakness. In addition, prednisone improves the gene expression and partially the protein expression of the dystroglycan [28]. Considering that dystroglycan is directly linked to dystrophin, we can suggest that prednisone has a potential effect on protein modulation of the skeletal muscle contractile apparatus. Interestingly in our study, the groups treated with either prednisone alone or combined with PBMT confirm these findings. Although the association of prednisone and PBMT has triggered improvement in most morphological aspects and protein expression of dystrophin, no improvement in functional performance evaluation was observed as expected. Thus, we suggest that the inhibitory effects of corticosteroids on PBMT [29] might have contributed negatively in this aspect.

Complications in muscle mechanics due to lack of dystrophin lead to a non-resolving inflammatory condition, culminating in deficits in tissue repair and consequent degeneration of skeletal muscle tissue [2, 30, 31]. Although there is some indication that NSAIDs may have similar effects to glucocorticoids on *mdx* mice [11], our results did not demonstrate these effects. Isolated use of NSAIDs did not appear to trigger significantly beneficial effects on dystrophic skeletal muscle tissue. Only when applied in association with the PBMT it was possible to observe improvement in the outcomes investigated, and this may suggest an isolated action of the PBMT and not to the association between both treatments. Since the outcomes of glucocorticoids were superior to NSAIDs, we can infer that the effects of glucocorticoids in DMD are not based exclusively on the anti-inflammatory action of these drugs, but also due immune system modulation.

The beneficial effects of PBMT on morphological, biomolecular and functional aspects observed in the present study corroborate with previous studies [9, 10]. Our results may suggest that the protective effects of PBMT on skeletal muscle tissue may have a positive impact on mechanical destabilization during the muscle contraction process. Thus, preventing DMD characteristics such as skeletal muscle injury followed by replacement of muscle tissue by fibrosis and adipose tissue [2]. In addition, or results confirm the ergogenic effects of PBMT on skeletal muscle [32, 33] previously demonstrated in healthy sedentary individuals [34, 35], athletes [36–39], or in some diseases [40–43], both in experimental studies [35] or randomized clinical trials [44–47]. In fact, PBMT has already demonstrated its ability to delay skeletal muscle fatigue [34, 35], increase exercise performance [34], protect skeletal muscle against damage [35], and decrease the effects of deconditioning [48]. Although muscle function was also improved with the use of glucocorticoid, PBMT proved to be more effective in this study.

It is important to highlight that prolonged use of glucocorticoids causes adverse effects in different organs and systems, requiring DMD patients to washout periods to minimize these undesirable effects [49]. On the other hand, the PBMT does not cause adverse effects and presented very interesting and promising results in the present study, as increased improvement in skeletal muscle function than glucocorticoids. Thus, we believe that PBMT has the potential to be considered a safe alternative for patients with DMD in a washout period, allowing them to remain receiving an effective and safe treatment during this period.

Thus, we believe that in a near future PBMT can be used as an adjunct therapy in the treatment of patients with DMD, but it is premature to say whether it could replace glucocorticoid therapy. Although our results are promising and optimistic, further studies will be needed to deepen the questions about PBMT’s mechanism of action and biological interactions in this disease. In addition, many clinical researches are obviously needed to confirm the effects observed in experimental research using animal models.

## Conclusion

In summary our results demonstrated that glucocorticoids and PBMT applied alone and in combination showed very positive results regarding the preservation of muscular morphology and to increase protein expression of dystrophin, however, there was not observed positive effects of this association in functional analysis. On the other hand, the treatment with NSAID applied alone not appears be an interesting alternative in *mdx* mice and only when associated with PBMT were achieved positive outcomes.

## Supporting information

S1 Dataset (PDF)

S2 Dataset (PDF)

S3 Dataset (PDF)
