# Supplementary material for: Can photobiomodulation therapy be an alternative to pharmacological therapies in decreasing the progression of skeletal muscle impairments of mdx mice?
Source: PLoS One. 2020 Aug 12;15(8):e0236689. doi: 10.1371/journal.pone.0236689 (PMC7423120; doi:10.1371/journal.pone.0236689)
Supplement: S1 Dataset — (PDF) [file pone.0236689.s001.pdf]

Number of fibers

WT	Placebo-control	PBMT	Prednisone	NSAID	PBMT + Prednisone	PBMT + NSAID
13	7	11	12	8	9	10
14	6	11	12	8	9	11
13	6	12	12	9	8	11
13	6	12	11	8	8	10
14	7	12	11	8	8	11

Centronuclear fibers

WT	Placebo-control	PBMT	Prednisone	NSAID	PBMT + Prednisone	PBMT + NSAID
1	18	4	12	16	6	10
0	16	4	13	18	6	11
0	19	6	12	18	6	10
1	17	5	11	17	6	12
0	18	5	12	17	7	11

Cluster nuclei

WT	Placebo-control	PBMT	Prednisone	NSAID	PBMT + Prednisone	PBMT + NSAID
0	8	2	7	15	8	7
0	10	1	8	12	9	9
0	8	2	8	12	9	9
0	9	2	9	13	9	11
0	9	2	8	13	8	9

Connective tissue

WT	Placebo-control	PBMT	Prednisone	NSAID	PBMT + Prednisone	PBMT + NSAID
181	788	199	165	2111	177	187
164	974	212	199	1878	201	232
208	878	195	183	1662	212	169
195	729	172	188	1869	188	193
186	843	194	184	1882	195	195

Fibers size

WT	Placebo-control	PBMT	Prednisone	NSAID	PBMT + Prednisone	PBMT + NSAID
61	41	51	55	44	44	52
59	39	51	55	44	44	53
58	39	56	54	45	45	55
59	38	57	57	47	46	54
60	42	55	53	41	45	54
57	40	55	55	43	44	56
60	40	57	56	43	45	53
60	39	54	58	44	46	55
61	40	55	52	41	43	55
60	40	54	52	43	43	53
57	40	53	54	43	45	55
58	38	54	54	41	45	53
58	39	58	56	44	44	53
61	38	55	57	43	44	56
60	37	58	53	45	44	52
60	40	58	55	45	44	57
56	41	57	55	42	46	55
62	38	57	58	44	46	55
60	38	55	56	41	45	54
60	37	55	56	42	44	57
57	41	53	54	43	46	54
58	40	56	53	43	43	53
57	38	57	53	45	44	53
60	37	52	55	41	43	55
62	39	53	55	41	44	55
63	40	53	56	41	44	55
56	40	52	54	41	45	56
58	43	52	55	43	43	52
61	39	53	56	44	46	53
60	38	53	53	41	42	55
60	40	53	55	43	44	54
61	44	51	53	44	44	56
58	37	52	54	45	44	54
	39	51	53	45	43	54
	39	50	56	44	45	57
		54	55	41	44	54
		52	56	42	45	55
		51	56	43	45	53
			54	44	45	57
			57	46	45	54
			57	47	45	
			55			
