# Supplementary material for: Can photobiomodulation therapy be an alternative to pharmacological therapies in decreasing the progression of skeletal muscle impairments of mdx mice?
Source: PLoS One. 2020 Aug 12;15(8):e0236689. doi: 10.1371/journal.pone.0236689 (PMC7423120; doi:10.1371/journal.pone.0236689)
Supplement: S2 Dataset — (PDF) [file pone.0236689.s002.pdf]

WT	Placebo-control	PBMT	Prednisone	NSAID	PBMT + Prednisone	PBMT + NSAID
107	26	67	61	30	64	60
87	20	75	52	12	70	56
111	35	76	63	32	67	77
94	28	61	51	20	59	57
83	29	54	57	24	50	46
