# Supplementary material for: Can photobiomodulation therapy be an alternative to pharmacological therapies in decreasing the progression of skeletal muscle impairments of mdx mice?
Source: PLoS One. 2020 Aug 12;15(8):e0236689. doi: 10.1371/journal.pone.0236689 (PMC7423120; doi:10.1371/journal.pone.0236689)
Supplement: S3 Dataset — (PDF) [file pone.0236689.s003.pdf]

Baseline

Placebo-control	PBMT	Prednisone	NSAID	PBMT + Prednisone	PBMT + NSAID
4	4	6	5	4	5
5	5	5	5	3	6
5	4	4	6	4	4
4	4	3	6	5	4
6	5	4	3	6	5

Post-treatments

Placebo-control	PBMT	Prednisone	NSAID	PBMT + Prednisone	PBMT + NSAID
5	20	6	5	8	1
3	26	13	12	3	12
5	21	14	5	4	7
3	22	23	3	10	8
4	37	9	8	3	3
